# Effects of therapeutic climbing in healthcare and rehabilitation: A systematic review protocol

**DOI:** 10.1371/journal.pone.0333796

**Published:** 2025-10-24

**Authors:** Bastien Delhoste, Fadl Moudni, Asiya Parvin Allaudeen, Bruno Chenuel, Edem Allado

**Affiliations:** 1 Groupe Hospitalier de la Région de Mulhouse et Sud Alsace (GHRMSA), Mulhouse, France; 2 CHRU-Nancy, Exploration Fonctionnelle Respiratoire – Centre Universitaire de Médecine du Sport et Activité Physique Adaptée, Nancy, France; 3 Rumb, Nancy, France; 4 Université de Lorraine, DevAH, Nancy, France; Lorestan University, IRAN, ISLAMIC REPUBLIC OF

## Abstract

**Background:**

Climbing is a fast-growing physical activity around the world. From recreational activity to sport, more and more people are practicing.

Initially used in the treatment of psychiatric and psychological disorders, climbing’s scope of application has expanded to include orthopedic, pediatric and neurological rehabilitation.

Nowadays, it is a therapeutic tool used by many physical and psychological rehabilitation professionals. The aim of this coming systematic review is to determine the therapeutic effects of climbing for promoting physical activity in healthcare and rehabilitation. We will identify the pathologies for which this tool is available, and relate the climbing protocols used to the effects obtained (tool performance and undesirable effects).

**Methods and analysis:**

We will include studies in which the effects of climbing or bouldering, used as a therapeutic tool, have been studied. The literature search will be conducted until 20 July 2025 using the databases MEDLINE, EMBASE, The Cochrane Library, Web of Science, and EBSCO. Following PRISMA guidelines, three reviewers in two groups will carry out all tasks: screening, selection and data extraction. The two groups of reviewers will assess each study’s risk of bias using ROB-2 for randomized clinical trial (RCTs), ROBINS-I for non-randomized clinical trial (CT) and the Joanna Briggs Institute Critical Appraisal tools for use in Systematic Reviews for case of reports/series and non-controlled clinical trials. Disagreement will be resolved by discussion until the reviewers reach consensus.

**Strenghts and limitations of the study:**

One of the strengths of this study is the description of the protocols used in the therapeutic climbing sessions, which has not been much reported until now. The analysis of the therapeutic effects on different dimensions is also a strength of this study. The main limitation of the study is that the results are likely to be biased according to the heterogeneity of the patients, protocols and measurement tools used in the selected studies.

## Introduction

Climbing is a worldwide fast-growing physical activity. Practiced as a recreational activity or a sport, more and more people are involved in this discipline [1].

Firstly listed as a Youth Olympic discipline since 2018, then an Olympic Sport since 2020, climbing is also on track to become a Paralympic discipline in 2028. There are almost 25 million practitioners around the world, in over 150 countries [2,3]. The fact that 60% of climbers are beginners is another indicator of this fast-growing activity [3].In France, the growth has been impressive, with the number of climbers tripling to 3 million between 2016 and 2022; for 10 million annual visits to climbing centers [4].

It is a physical activity that brings into play many elements, whether physical, cognitive, visual or even psychological aspects. In addition to the physical activity itself, it enables you to organize yourself, overcome difficulties, manage your emotions, concentrate, perceive your environment and become aware of your body and its spatial position [1,5].

Based upon this whole-body work-out encompassing mental and social health determinants, the idea of using climbing as a therapeutic tool was rapidly born [6]. Therapeutic climbing (TC) was first used to treat psychological and psychiatric pathologies in the 1980s [6]. Earliest studies involved patients with post-traumatic stress disorder, depression, anxiety and autism. In the 21st century, with the worldwide explosion of this practice, the use of therapeutic climbing has been diversified in various diseases as stroke, multiple sclerosis, low back pain or Parkinson’s disease [7]. Numerous studies have been carried out to assess the therapeutic efficacy of this tool on patients suffering from these chronic diseases [8,9]. These studies have shown significant improvements in balance, muscular strength, coordination and mobility in patients who have benefited from TC sessions [7]. This also makes it an excellent means of inclusion and for promoting physical activity. Indeed, giving everyone the chance to take part in physical activity is a major asset in current policies to manage chronic diseases and combat sedentary lifestyles [10]. Then TC is currently used as a therapeutic tool in rehabilitation and healthcare, demonstrating positive effects in various fields of application, including neurology, orthopedics, psychiatry and psychology [7,11–17].

However, TC involve a large spectrum of practices. It could be bouldering without a rope, or lead climbing, rock or indoor wall climbing. In addition to these different forms of climbing, we can also observe that there is no international consensus on the way to carry out a TC session: with or without an instructor, in a group or individually, type and duration of the practice (short or full-day sessions). These techniques all require the presence of a specially trained professional to guide the session and ensure safety. This can be an obstacle, with a limited number of these professionals, a higher cost or even a scattered distribution of these professionals over the country [18]. These factors also add to the apprehension of participants or prescribers. Studies show, however, that this is a safe practice, with an injury rate of 0.02 injuries per 1000 hours of practice, and can be performed by anyone, whatever their age or chronic disease [19,20].

A systematic review and meta-analysis performed in 2021 demonstrated the beneficial effects of using TC on certain criteria in the care management of various pathologies [7]. However, these conclusions should be treated with caution, given the small number of studies available at the time of selection, and the methodological weaknesses of some of the included studies. Indeed, in the current literature there are serious variations in the duration or length of intervention, frequency of the training sessions and protocols used in TC [8,9,11–15,17,18]. At the same time, the use of climbing in healthcare or as a therapeutic tool has considerably increased in recent years, which means that this knowledge needs to be regularly updated. A narrative review published in 2022 reports the use of therapeutic climbing in 3 main pathologies: multiple sclerosis, depression and chronic low back pain [6]. However, this review focuses little on the protocols used in the various studies, but rather on the origin of therapeutic climbing and the mechanisms at play during these sessions.

To summarize, TC is an increasingly tool used in healthcare and rehabilitation for a very wide public, with a certain number of positive effects being demonstrated [7]. The question is therefore: what are the different protocols used for the use of climbing in rehabilitation and health, and what are the therapeutic effects of these sessions? We will therefore determine the therapeutic effects and protocols used, and describe the conditions under which this tool can be used as a treatment. We assume that TC is a safe and effective tool for supporting diverse populations in the practice of physical activity for health purposes [6]. It can also be associated with a rehabilitation program for patients of all ages, suffering from various pathologies.

### Review question

What are the physical, cognitive and social effects of using climbing as a therapeutic tool with children, adolescents and adults?

### Objectives

This systematic review aims:

- To determine the physical, cognitive or social effects of therapeutic climbing sessions used in health or rehabilitation.
- to determine the different protocols (duration, frequency, equipment) used according to the pathologies targeted for the use of this tool..

## Methods

### Principles

The Preferred Reporting Items for Systematic Review and Meta-Analysis Protocol (PRISMA-P) will be used to report this protocol [21]. The PRISMA-P checklist is attached as online supplemental file 1.

This review protocol was registered at the International Prospective Register of Systematic Review (PROSPERO).

Registration number: CRD42024557040.

### Eligibility criteria

This systematic review will include articles corresponding to the following PICOS scheme (population, intervention, comparison, outcome and study design).

#### Types of participants.

Our systematic review will focus on children, adolescents and adults with health concerns who use climbing as a therapeutic tool.

#### Types of interventions.

Studies exploring the effects of therapeutic climbing on patients will be included in this study. The same will be true for studies investigating the use of bouldering in patients, which do not include psychotherapy during these sessions.

#### Types of comparison.

In order to be able to examine the different therapeutic effects of climbing, all studies, with or without a control group, will be studied.

#### Types of outcomes.

We will be looking at the therapeutic effects on the physical aspect (e.g., muscle strength, pain or balance), the social aspect (e.g., social interaction) and the cognitive and mental aspects (e.g., anxiety, depression or self-esteem).

The way in which the sessions are structured, the equipment used and the guidance provided will also be points of interest.

### Search strategy for identifying relevant studies

Following PRISMA guidelines, two reviewers will carry out all tasks: screening, selection, data extraction. In case of disagreement, discussion and consensus between the reviewers will be followed.

#### Bibliographic database searches and other sources searches.

The literature search will be conducted until 20 July 2025 using the databases MEDLINE, EMBASE, The Cochrane Library, Web of Science, and EBSCO. The main terms to be used are ‘climbing’, ‘bouldering’, ‘therapeutic’, ‘therapy’ or ‘physical activity’. The following Table 1 shows the search equation that will be used to collect articles from the EMBASE database.

**Table 1:** Systematic review -research equation.

#1	‘climbing’ OR ‘bouldering’
#2	‘Health’ OR ‘Therapeutic’ OR ‘Therapy’ OR ‘Therapies’ OR ‘Treatment’ OR ‘Treatments’ OR ‘health-related fitness’ OR ‘fitness’ OR ‘exercices’ OR ‘exercice’ OR ‘physical activity’ OR ‘physical activities’ OR ‘physical education’
#3	#1 (intervention) AND #2 (outcome) (‘climbing’ OR ‘bouldering’) AND (‘Health’ OR ‘Therapeutic’ OR ‘Therapy’ OR ‘Therapies’ OR ‘Treatment’ OR ‘Treatments’ OR ‘health-related fitness’ OR ‘fitness’ OR ‘exercices’ OR ‘exercice’ OR ‘physical activity’ OR ‘physical activities’ OR ‘physical education’)

The references of all relevant publications will be examined for additional relevant information sources missed during our search and full texts will be retrieved. References of relevant reviews will also be examined.

#### Study design.

We will include randomized controlled trials, controlled clinical trials, non-controlled trials and case studies to assess the effects of the intervention and will supplement these with observational studies. Systematic reviews and meta-analyses will not be included.

### Selection of studies

All studies that might be relevant will be imported into the Rayyan software (https://www.rayyan.ai), and duplicates will be eliminated. Two reviewers will independently screen the titles and abstracts for inclusion, using Rayyan software and following the previously established criteria. Any disagreements will be settled through discussion between the reviewers to reach a consensus. Authors of publications for which the full text is not available online will be contacted by e-mail in order to obtain the publication. The reviewer will send two reminders at three-week intervals. If there is no response, these studies will be excluded from this systematic review. The same applies to studies published in a language other than English, French or German. Subsequently, two reviewers will independently examine the full texts of all potentially eligible studies, compare their findings, and resolve any discrepancies through discussion. Inter-rater agreements between investigators for study inclusion will be assessed using Cohen’s κ [22].

### Data extraction and management

The two groups of independents reviewers will collect information about the author, year of publication, country, study design, size of the population, characteristics of the population (age, disease, gender), eventual a comparison group, protocol used for therapeutic climbing (therapists, number of participants, number of sessions, duration, frequency, intensity, etc.), functional, cognitive or psychological therapeutic effect obtained (inferential statistic test and/or size effect test). Adverse events (injury, for example) noted by the authors of the various studies will also be reported.. Studies in which relevant data are impossible to extract will be excluded. In case of disagreement, discussion and consensus between the reviewers will be followed.

### Treatment of missing data

In instances where essential information is missing from included studies (e.g., participant characteristics, intervention details, or primary outcomes), the corresponding authors will be contacted to obtain clarification. If the required information cannot be retrieved, the missing data will be explicitly reported in the review and accounted for in the interpretation of the findings. No statistical imputation will be performed due to the absence of a meta-analysis. Additionally, the potential impact of missing data on the quality and robustness of the interpretation will be critically assessed within this systematic review.

### Assessment of risk of bias

Two independent reviewers will assess the included studies for quality, internal validity, and risk of bias, using the Cochrane risk-of-bias tool for for a rigorous and detailed assessment of the risks of bias in the conduct of randomised controlled trials ROB-2, the Risk Of Bias In Non-Randomized Studies – of Interventions (ROBINS-I) and the Joanna Briggs Institute Critical Appraisal tools for use in Systematic Reviews for case of reports/series and non-controlled clinical trials [23–25]. In case of disagreement between the reviewers, it will be resolved through discussion.

### Presentation and reporting of results

The study selection process will be summarized using a flow diagram.

In this review, after extracting data such as author, year of publication, country, study design, population characteristics, and sample size, we will first compile information regarding the different protocols employed during the climbing sessions. We will then document the supervisors’ qualifications, group sizes, wall dimensions and inclination, instructions provided during sessions, routes followed, as well as the duration and frequency of the interventions. Finally, the therapeutic outcomes will be categorized and compared according to their physical, cognitive, or social effects.

### Patient and public involvement

Patients and the public were not involved in the design or planning of the study.

### Potential amendments

We do not plan to modify the protocol to avoid reporting bias. However, if necessary, any amendment in the review process will be reported for transparency.

### Ethics and dissemination

As no primary data will be collected in this study, no ethical approval is required. This review aims to evaluate the accuracy of digital tools by evaluating both quantitative and qualitative indicators for monitoring wound healing, and to offer a detailed description of the devices. The final report will be published in a peer-reviewed journal.

### Timeline expectation

Bibliographic database searches (July 2025), selection of included studies (July 2025), data extraction and management (August 2025), data synthesis and analysis (September 2025), manuscript submission (November 2025).

### Patient consent for publication

Not required

## Conclusion

The aim of this systematic review is to study the therapeutic effects of climbing on patients, and to identify the different protocols used during climbing sessions. This is a broad objective for a systematic review, but it is necessary to establish the current state of the art by presenting the most robust methodology possible. The main limitation of the study is that the results are likely to be biased according to the heterogeneity of the patients, protocols and measurement tools used in the selected studies

## Supporting information

S1 File PRISMA-P 2015 Checklist. (PDF)
